# Draft Genome Sequences of *Acinetobacter baumannii* Strains Harboring the *bla*_NDM-1_ Gene Isolated in Lebanon from Civilians Wounded during the Syrian Civil War

**DOI:** 10.1128/genomeA.01678-15

**Published:** 2016-01-28

**Authors:** Sima Tokajian, Jonathan A. Eisen, Guillaume Jospin, Monzer Hamze, Rayane Rafei, Tamara Salloum, Joe Ibrahim, David A. Coil

**Affiliations:** a Department of Natural Sciences, Lebanese American University, School of Arts and Sciences, Byblos, Lebanon; b University of California Davis Genome Center, Davis, California, USA; c Health and Environmental Microbiology Laboratory, Azm Centre for Biotechnology, Doctoral School for Sciences and Technology-Lebanese University, Beirut, Lebanon

## Abstract

We present here the draft genome sequences of multidrug-resistant *bla*_NDM-1_-positive *Acinetobacter baumannii* strains ACMH-6200 and ACMH-6201, isolated in north Lebanon from civilians wounded during the Syrian civil war. The draft genomes were contained in 217 contigs for ACMH-6200 and 83 contigs for ACMH-6201, including a combined 3,997,237 bases for ACMH-6200 and 3,983,110 bases for ACMH-6201, with 39% and 38.9% G+C content, respectively.

## GENOME ANNOUNCEMENT

*Acinetobacter baumannii* has gained much importance in recent years for being a major cause of hospital-acquired infections worldwide, especially in developing countries (1). Successful infection with the pathogen is primarily achieved via breaks in the skin in case of wounds or burns, or through a compromised respiratory system (2, 3). Other areas where *A. baumannii* strains are of high concern are military field hospitals and field hospitals erected in the wake of natural disasters (4). In both locales, being an opportunistic pathogen (5), *A. baumannii* is manifested particularly in burn, open wound, and trauma patients, and in patients requiring mechanical ventilation (5–7). Multidrug-resistant *A. baumannii* is rapidly emerging as an important pathogen in the health care setting. This low-virulence organism, which survives for prolonged periods in the environment and under unfavorable conditions, disseminates antibiotic resistance by virtue of its extraordinary ability to accept and donate resistance plasmids (8). *A. baumannii* strains are resistant to desiccation, disinfectants, and nutritional starvation, all of which are ideal for the organism to thrive on both moist and dry surfaces in the nosocomial environment (9). Moreover, *A. baumannii* strains have been known to form biofilms, which further enhance their ability to colonize and spread on inanimate surfaces (8). The resistance among *A. baumannii* strains to almost all classes of antimicrobial agents, and especially to β-lactams and particularly to carbapenems, is on the rise (10). Carbapenem resistance due to the production of metallo-β-lactamases, such as the New Delhi MBL (NDM), is becoming a serious health care concern (8).

In this study, we sequenced *A. baumannii* ACMH-6200 and ACMH-6201, which harbor the *bla*_NDM-1_ gene, and both were recovered in Lebanon from Syrian patients wounded during the civil war (11).

A Nextera XT kit (Illumina, San Diego, CA, USA) was used to simultaneously fragment and adapter tag the library, as per the manufacturer’s instructions. The library was normalized by bead-based affinity and then sequenced using the MiSeq version 3 600-cycle kit (Illumina) to perform 300-bp paired-end sequencing on a MiSeq instrument (Illumina), as per the manufacturer’s instructions. Quality trimming and error correction of the reads resulted in 665,598 high-quality reads for ACMH-6200 and 1,684,290 reads for ACMH-6201. Sequence processing and assembly were performed using the A5-miseq assembly pipeline. This pipeline automates the processes of data cleaning, error correction, contig assembly, scaffolding, and quality control (12, 13). The initial assembly produced 217 contigs for *A. baumannii* ACMH-6200 and 83 contigs for ACMH-6201, for which no scaffolding was obtained. The final collection of contigs was submitted to GenBank. The final draft genome sequences consisted of a combined 3,997,237 bases for ACMH-6200 and 3,983,110 bases for ACMH-6201, with 39% and 38.9% G+C content, respectively. Automated annotation was performed using the RAST server (14). *A. baumannii* ACMH-6200 contains 3,766 predicted coding sequences and 77 predicted RNAs, while ACMH-6201 contains 3,778 predicted coding sequences and 78 predicted RNAs. Moreover, the ResFinder 2.1 Web server revealed that both isolates additionally harbored the *bla*_OXA-94_ gene (https://cge.cbs.dtu.dk/services/PlasmidFinder/) (15).

### Nucleotide sequence accession numbers.

These whole-genome shotgun projects have been deposited at DDBJ/EMBL/GenBank under the accession numbers LKMA00000000 for ACMH-6200 and LKMB00000000 for ACMH-6201. The versions described in this paper are the first versions.
